# The National Pension Fund for Nurses

**Published:** 1888-10-20

**Authors:** 


### The National Pension Fund for Nurses.

There was a large attendance of ladies and gentlemen, including many nurses, on Wednesday evening, the 10th inst., in the Governors' Hall of St. Thomas's Hospital, when Mr. Edward T. Clifford, the Hon. Manager of the National Pension Fund for Nurses, read a paper upon the Fund. Dr. Bristowe, F.R.S., occupied the chair.

Dr. Bristowe briefly introduced Mr. Clifford, the substance of whose paper we published last week (copies may be had on application to 8, King Street, E.C.), and expressed pleasure at seeing such a large and representative gathering. The subject of the National Pension Fund for Nurses was one of great interest and of very great importance, and he anticipated an interesting paper and a valuable discussion.

The points of the paper were warmly cheered, and Mr. Clifford concluded amid much applause.

Mr. Henry C. Burdett, at the request of the Chairman, opened the discussion. He said he desired to point out one thing which had struck him rather forcibly that afternoon, when he called at the offices of the Fund in order to ask one or two questions. Mr. Clifford, the honorary manager, was there, and was evidently suffering from the depression which must necessarily overtake the novice in nursing matters, who was unaware of the immense amount of distress which existed at present amongst the older nurses of this country. He (Mr. Burdett) had been engaged for twenty years and more with nurses—sometimes working side by side with them, sometimes acting as their master, and sometimes helping them to take up nursing—and during that period he had been grieved by the sad condition to which all classes of the nursing profession, superintendents and nurses alike, were sometimes reduced, after having devoted the best portion of their lives to the care of others; and he had been greatly shocked to see them, in so sad a plight, at the very period of their lives when they were least able to take care of themselves. Well, Mr. Clifford that afternoon was suffering from the shock arising from the distressing applications he had received from nurses during the six or eight weeks he had had to take charge of the Fund. What the promoters of the Fund had to do was to try to take care that from the date of its establishment, no nurse who joined the Fund—however small her contribution to it might be, so long as it represented what she was justified in putting by, —should be left destitute, without comfort and without help when she found herself either out of work or in ill health. If that were done, it would be possible, before many years were gone by, to say to nurses who were in distress, and had made no provision for it "We did our utmost to induce you to join the Fund; you have been warned, you have seen what the effect of the want of provision has been upon those with whom you have worked who have become old, and you have failed to take advantage of these opportunities; therefore the full sympathy which we should otherwise give you must be tempered." Nurses seem to have an idea in regard to the Fund, that they had got to find a table which would produce at once on paper in return for what they could save year by year, exactly the retiring allowance, which they justly considered they ought to get at a certain age. He ventured to think this was entirely an erroneous way of looking at the matter. The career of nursing was like every other career, a progressive one, and there were several grades, with different emoluments, that is to say, the probationer, nurse, sister, night superintendent, and matron. Thus all those who were probationers should begin with their shilling a-month, bravely and cheerfully, while the nurses who had at the present time heavy claims upon them from relatives or others, could, and indeed, must, save at any rate a similar sum every year. If they placed those savings in the Fund they could, by paying in under the returnable scale, use it as a savings bank, and enable the directors of the Fund to take an interest in them, and help them to the utmost of their power. It must be understood that the National Pension Fund for Nurses did not profess or hope to provide tables which would show every nurse in the country how the exact amount of savings which she could put by to-day would produce the sum which she required and thought she ought to have when she retired from nursing altogether. All the directors of the Fund said to nurses was: "Do your best; do something; and we will look after your interests as if they were our own. If you do not do this the responsibility—and it is an awful responsibility—for the future must rest entirely, from this time forth, upon you, and upon every other individual nurse who does not choose to join."

He should like further to point out, *àpropos* of this, that although the objects of the Fund were mainly to provide pensions for nurses, the directors had the right to make semi-gratuitous payments under certain conditions in deserving cases to those who had joined the Fund. It must also be remembered that many donors to the Bonus Fund and many of those who were wishing to give, but whom the directors desired should not give at this stage, hoped to give specially with a view to the bonus being divided to help those nurses whose payments though small, because they were the poorest, had probably been earned by work which was the hardest. Nurses might be perfectly certain of this, that those who incepted the Fund had made the constitution so wide, that it really embraced everything which could be useul to the nurses. The articles of association even included provision for establishing a register in case the other organizations which were attempting to institute a register for nurses should not be able to carry their scheme through to success. The Fund itself thus had the power, although it had no desire or intention unless it was compelled to exercise it, to establish a register, and to take care that even that branch of the work was not neglected or allowed to fall to the ground. Nurses might be perfectly certain that if they joined this Fund they would eventually get—(1) interest for their money larger than they could get anywhere else; (2) membership of an organisation conducted by those whose one desire was to comfort and sustain and help nurses. He desired to say further that there was now no possibility of the £26,000 being returned to the original donors, for if those who had already taken out policies would each bring one friend to join also, a thousand policies would certainly be issued within the first three months of next year.

He had taken great pains to ascertain the facts, and counting all classes of nurses of every degree, he believed there were not more than 15,000 nurses in this country, and of these probably not more than 10,000, if so many, were at present actively engaged and able to join the Fund. It would be an enormous advantage educationally to the whole body of nurses, when one out of every ten in active work had proved, by joining the Pension Fund, that they wished to help their best friends to help them to an adequate provision. Many people had said it was quite impossible for the nurses to save anything, and that sufficient alms or money must be raised to give every nurse who would accept charity, as much as she required. To this the National Pension Fund directors replied, if that was the position of the nurses, it must be wrong to talk about the profession of nursing, and that if the nurses had not sufficient provident instinct in them to pay a few shillings into a fund like this, it was quite clear that no good would be done to nursing if the conditions upon which the £26,000 had been given were not adhered to. Holding these views it had been determined at the outset that the National Pension Fund must be wound up if the one-thousand nurses were not forthcoming in two years time. He himself was very strong upon this point, and he was sure that all in that room would agree with him, that they could not merely offer charity to nurses, and must feel that it would be a degradation to do anything of the kind. It was, however, proposed to let those who had benefited by nurses' work, and who desired to show their thankfulness, spontaneously give such contributions as they might think fit to the Bonus Fund from time to time. The applications were very numerous at present, and the staff were actively engaged from morning till night. He only hoped that all those who heard him would take an early opportunity of assuming the proud position in the eyes of the whole country, which a few months would render it impossible for them to occupy. This position might be secured by all who join the Fund immediately, because they would then be one of the first one thousand nurses who, at the time when nursing was put to the test, came forward and said: "We believe in nursing as an honourable profession; we believe in the elevation of nursing; we decline altogether to show by hanging back at the outset that we wish for alms; all we desire is a reasonable amount of assistance, for services rendered, from grateful hearts; and then we ourselves shall be able to do the rest." I appeal to every nurse, individually, to ensure by taking this step, that for the future, owing to the National Pension Fund and her co-operation with its managers, no nurse, when she is sick or when she retires from service, need be without a powerful friend able to afford her adequate and proper provision. (Loud cheers.)

Mr. King, the Actuary to the Fund, remarked upon the controversy with the *Lancet*, and suggested, amid cheers, that the *Lancet* had got the worst of it. The *Lancet*, he said, had made a great deal of the point as to why the founders of the Fund did not reduce their rates. The reason was this. There was nowadays a certain and steady fall in the rates of interest, and it must be remembered that those responsible for the Fund had to invest the money for the next ten and fifteen and many more years. They were therefore bound to be on the safe side. Again, the rate of mortality in the country was falling, and that meant that more people would live to draw deferred annuities, and those who lived to draw them would live longer and draw more. Taking these two points into consideration, he had come to the conclusion that they could not lower the rates; but the effect of their caution was this, that as the Association was a mutual one, any surplus would go to increase the annuities, so that if they had been too cautious, they would give more than they promised, and if they were not, they would only keep their promise, and no man was bound to do more than that. (Applause.) A lady had written to him, hoping that length of service would be recognised. In one sense that was impossible, because the actuary could not be benevolent, and a nurse was not likely to live any shorter because she had been twelve years a nurse than one who had only been a nurse a year, and if the two joined at the same age the actuary could not make any difference. It was, however, possible that length of service might be taken into consideration through the bonus fund. What Mr. Burdett had said about distress amongst the older nurses pointed clearly to the necessity of the Fund.

Mr. Ryan (St. Mary's Hospital) urged upon nurses to lay their case before the committees of their respective hospitals, whom he had no doubt would render them willing aid in bearing a proportion of the charge of the premiums. He explained how a meeting had been held at his hospital in connexion with the Fund, and how he was able, after careful examination, to assure the nurses that the advantages offered by the Fund were greater, and quite as certain, as those which were provided by the Post Office. He said his governors would be only too glad to co-operate with the nurses who wished to join it. Let them memorialize their committees without a moment's delay, and they would be helped most cheerfully he was certain. He expressed surprise that nurses had not taken action in the matter already for themselves, and offered the advice to them to do so, as—what he termed amidst laughter—a "tip."

Dr. George W. Potter said that there had been that evening a good deal of what the Prayer-book termed "commination." One reason why some nurses did not take up the Fund with enthusiasm was that they were not so selfish as men; they did not look so far forward and say to themselves, "What is going to happen to me when I am 50 years of age?" He was surprised, considering all the opposition, that there had been so many applying. He wished to press upon the nurses the fact that this Fund was not Mr. Burdett's fund, or Mr. King's, or Mr. Clifford's, but their Fund, and they must be a little more selfish in looking after their own interests. They were all resolved that the Fund should be an adequate Pension Fund for everybody who joined it. He knew Mr. Clifford was determined, by hook or by crook, to carry his way in that respect. They must all become missionaries for the Fund, and then they would make it a grand success.

Dr. Bristowe, F.R.S., commented upon the excellent paper which had been read, and remarked that he almost regretted it had not led to some adverse criticism. The offer to nurses which was made by the present Fund was almost unique, and if it were to fall through the offer was not likely to be made again. Nurses should certainly endeavour to subscribe to it, for the Fund was established on a thoroughly substantial basis, and would, he had not the slightest doubt, be a thorough and deserved success. He concluded by proposing a vote of thanks to Mr. Clifford for his paper

The vote was carried by acclamation, and Mr. Clifford briefly responded, proposing at the same time a similar compliment to the chairman. Mr. Burdett seconded the motion, and paid a warm tribute of admiration to Dr. Bristowe. He also wished it to go forth to the country that when the council found it neccessary to engage the services of officers with a sound knowledge of insurance, they took special steps to find the best man for the post of manager, and they were confident that Mr. Clifford would soon show by results that he was that man, and that the opinion of his abilities which Mr. King and others who knew his work had expressed would be more than justified. A resolution of thanks to the governors of St. Thomas's Hospital for the use of the hall, brought the proceedings to a close.

